# The Effects of Preoperative Oral Carbohydrate on Frequency of T and NK Cells in Patients with Cervical Cancer Treated Using Neoadjuvant Chemotherapy and Surgery: A Prospective Cohort Study

**DOI:** 10.1155/2020/2101480

**Published:** 2020-03-16

**Authors:** Fuqing Zhang, Mengxia Yao, Zhiping Lin, Yili Chen, Hui Jiang, Meina Zeng, Wenhua Chen

**Affiliations:** ^1^Department of Anesthesiology, Fujian Cancer Hospital & Fujian Medical University Cancer Hospital, China; ^2^Department of Anesthesiology, Fujian Medical University Union Hospital, China

## Abstract

**Background:**

Immune dysfunction can occur after neoadjuvant chemotherapy (NAC) and surgery for cancer. We investigated whether preoperative oral carbohydrate affected the postoperative percentages of T cells (CD4^+^ and CD8^+^) and natural killer (NK) cells in patients with cervical cancer treated with NAC and surgery.

**Methods:**

This prospective cohort study enrolled consecutive patients with cervical cancer treated by radical hysterectomy with PLND at the Gynecologic Oncology Department of Fujian Provincial Cancer Hospital (China) between January 2018 and December 2018. Patients were divided into three groups according to the treatment method: NAC (two cycles, surgery 1 month later), NAC+CHO (chemotherapy and surgical methods same as with the NAC group but with 300 mL of oral carbohydrate administered 2 h before surgery), and non-NAC (surgery alone). Percentages of NK, CD3^+^, CD4^+^, and CD8^+^ cells were evaluated by flow cytometry the day after the first admission, just before surgery, immediately after tracheal tube removal, and the day after surgery. This trial is registered with NCT03872635 at clinicaltrials.com.

**Results:**

The final analysis included 77 patients (non-NAC group, *n* = 26; NAC group, *n* = 25; and NAC-CHO group, *n* = 26). Baseline characteristics and preoperative NK, CD3^+^, CD4^+^, and CD8^+^ cell percentages were similar between groups. Postoperatively, all groups exhibited reductions in NK, CD3^+^, and CD4^+^ cell percentages and increases in CD8^+^ cell percentages (all *P* < 0.05). The changes in NK, CD3^+^, CD4^+^, and CD8^+^ cell percentages were attenuated in the NAC-CHO group (*P* < 0.05 vs. both other groups).

**Conclusion:**

Preoperative oral carbohydrate can improve the postoperative populations of NK and T cells after the treatment of cervical cancer by NAC and surgery.

## 1. Introduction

Cervical cancer is the fourth most common malignancy in females [1]. In 2015, the incidence rate of cervical cancer in China was 9.89% [2]. Therapies for cervical cancer include radical hysterectomy, chemotherapy, radiotherapy, and concurrent chemoradiotherapy [3]. However, the recurrence rate is 10%-50%, and the 5-year survival is around 65%, although this varies with the disease stage [4].

Radical hysterectomy (RH) with pelvic lymph node dissection (PLND) is one treatment option recommended by the National Comprehensive Cancer Network (NCCN) guidelines for stage IB and IIA cervical cancer [5]. Preoperative neoadjuvant chemotherapy (NAC) can reduce tumor size and facilitate surgical resection [6]. NAC results in a response in 53–79% of patients with cervical cancer [7–9], and its reported clinical benefits include improved surgical feasibility, reduced need for postoperative chemoradiation therapy, and longer progression-free survival and overall survival [10, 11].

The suppression of normal immune responses by cancer cells is an important mechanism contributing to tumor growth and proliferation [12], and there is increasing interest in the roles of lymphocytes such as CD4^+^ cells (helper T cells and regulatory T cells), CD8^+^ cells (cytotoxic T cells), and natural killer (NK) cells. Helper T cells facilitate other lymphocytes and activate macrophages and cytotoxic T cells. Regulatory T cells inhibit T cell-mediated immunity and are involved in immunologic tolerance. Cytotoxic T cells and NK cells can target and destroy tumor cells. Previous studies of patients with cervical cancer have described alterations in T cell and NK cell populations [13], suppression of NK cell activity by CD4^+^ regulatory T cells [14], and reduced numbers of CD4^+^ T cells and NK cells in the neoplastic cervix [15]. Importantly, RH for cervical cancer has been reported to decrease CD4^+^ T cell and NK cell numbers and the CD4^+^/CD8^+^ ratio [16], indicating that surgery can cause immune dysregulation. Although NAC for cervical cancer appears not to suppress lymphocyte numbers, lower levels of CD4^+^ T cells, CD8^+^ T cells, and NK cells are associated with a poorer response to chemotherapy [17, 18]. Additionally, polychemotherapy may inhibit NK cell activity without reducing NK cell numbers [19].

Reduced calorie intake has been shown to decrease CD4^+^ and CD8^+^ cell numbers in patients with rheumatoid arthritis [20], suggesting that preoperative fasting may contribute to the decline in lymphocyte numbers after surgery. There is increasing interest in the potential benefits of administering high-carbohydrate drinks before surgery. Oral carbohydrate intake rather than fasting before surgery can suppress postoperative metabolic disturbances such as insulin resistance, improve postoperative cardiac function, enhance postoperative patient comfort, and shorten hospital stay without increasing the incidence of complications [21–28]. Additionally, the preoperative oral carbohydrate may help to ameliorate the decline in T lymphocytes and NK cells following surgery [23].

We hypothesized a preoperative oral carbohydrate drink would attenuate postoperative immune system disturbances in patients treated for cervical cancer using NAC and surgery. The aim of this study was to investigate whether preoperative oral carbohydrate would attenuate postoperative changes in CD4^+^ T cell, CD8^+^ T cell, and NK cell numbers in patients with cervical cancer undergoing RH and PLND after NAC.

## 2. Material and Methods

### 2.1. Study Design and Participants

This prospective cohort study enrolled consecutive patients with cervical cancer treated by RH and PLND at the Gynecologic Oncology Department, Fujian Provincial Cancer Hospital, Fujian Medical University, Fuzhou, Fujian, China, between January 2018 and December 2018. The inclusion criteria were as follows: (1) female aged 18-60 years; (2) American Society of Anesthesiologists (ASA) level I or II; (3) cervical squamous cell carcinoma confirmed by cervical biopsy and pathology; (4) no distant metastasis observed on computed tomography (CT); (5) CT, magnetic resonance imaging (MRI), positron emission tomography (PE), and/or B-ultrasound and gynecologic examinations indicated stage IB1 or IIA1 disease with a cervical tumor mass of 2–4 cm (non-NAC group) or stage IB2 or IIA2 disease with a cervical tumor mass > 4 cm (NAC group); (6) the planned surgery was laparoscopically assisted RH with bilateral salpingo-oophorectomy and PLND; and (7) the planned chemotherapy regimen (NAC groups) was two courses of paclitaxel and cisplatin, with an interval of 4 weeks between the second course and surgery. The exclusion criteria were as follows: (1) previous chemotherapy (for patients in the NAC groups); (2) dysfunction of gastric emptying; (3) diabetes mellitus or impaired glucose tolerance; (4) body mass index (BMI) > 32 or <18.5 kg/m^2^; (5) multiple cervical tumors; (6) tumor recurrence; and (7) functional disorders of the respiratory, circulatory, immune, nervous, or urinary system or any other organs. Additionally, patients were excluded from the final analysis if they received a blood transfusion or nutritional support within the 2 weeks before surgery or were given a carbohydrate-containing solution, blood transfusion, glucocorticoid, or ephedrine (to treat hypotension) during surgery.

This study was performed in accordance with the principles of the Declaration of Helsinki and approved by the ethics committee of Fujian Medical University (KT2008-003-01). All patients provided informed written consent before enrolment. This study is registered at clinicaltrials.com (NCT03872635).

### 2.2. Patient Grouping

Patients were divided into three groups using a computer-generated random number table: the NAC group (stage IB2/IIA2 disease and a cervical tumor mass > 4 cm) treated using NAC followed by RH/PLND; the NAC+CHO group (stage IB2/IIA2 disease and a cervical tumor mass > 4 cm) treated using NAC, preoperative oral carbohydrate, and then RH/PLND; and the non-NAC group (stage IB1/IIA1 disease and a cervical tumor mass of 2–4 cm) treated by RH/PLND alone. Neither the participants nor the investigators were blinded to the intervention.

### 2.3. NAC

The patients in the NAC and NAC+CHO groups received two cycles of NAC. The 3-week chemotherapy regimen consisted of paclitaxel (135–175 mg/m^2^) and cisplatin (75 mg/m^2^). The interval between the end of the second cycle and surgery was 4 weeks.

### 2.4. Preoperative Preparation

All patients undertook preoperative bowel preparation. Patients in the non-NAC and NAC groups underwent an 8-hour preoperative fast for solids and a 4-hour preoperative fast for fluids. Patients in the NAC+CHO group underwent an 8-hour preoperative fast for solids, consumed 300 mL of an oral carbohydrate drink 2 hours before surgery, and then underwent a 2-hour preoperative fast for fluids. The main components of the carbohydrate drink (Shuneng multifunctional beverage, Yichang Renfu Pharmaceutical Co., Ltd.; batch number: S20160629) were carbohydrate, sodium, vitamin B1, vitamin B6, vitamin B12, zinc, and taurine.

### 2.5. Surgery

All operations started between 09:00 and 09:30 and were conducted by the same team of anesthesiologists and surgeons. Anesthesia was induced using midazolam (0.5 mg/kg; Jiangsu Enhua Pharmaceutical Co., Ltd.), fentanyl citrate (4 *μ*g/kg; Yichang Renfu Pharmaceutical Co., Ltd.), propofol (2 mg/kg; B. Braun Melsungen AG), and rocuronium bromide (8 mg/kg; Huabei Pharmaceutical Co., Ltd.). After tracheal intubation, general anesthesia was maintained using inhaled sevoflurane (1–2%; Shanghai Hengrui Pharmaceutical Co., Ltd.), propofol (2–5 mg/kg/h), remifentanil hydrochloride (0.05–0.15 *μ*g/kg/min; Yichang Renfu Pharmaceutical Co., Ltd.), and cisatracuriumbesylate (2–3 *μ*g/kg/min; Zhejiang Xianju Pharmaceutical Co., Ltd.) with a tidal volume of 6–8 mL/kg, a respiratory frequency of 10–12 times/min, and an end-expiratory carbon dioxide partial pressure of 35–45 mmHg. Intraoperative hypotension (blood pressure fall > 30% of the baseline value), hypertension (blood pressure rise > 30% of the baseline value), bradycardia, and tachycardia were managed by an intravenous injection of 10 mg ephedrine, 5 mg urapidil, 0.5 mg atropine, and 10 mg esmolol, respectively. Patients administered with ephedrine intraoperatively were excluded from the final analysis.

All patients underwent laparoscopically assisted RH with bilateral salpingo-oophorectomy and PLND. Postoperatively, the patients were fasted for 24 hours and given amino acids, fatty acids, and glucose for parenteral nutritional support. Postoperative analgesia involved the intravenous infusion of a solution (100 mL) containing 150 *μ*g sufentanil citrate (Yichang Renfu Pharmaceutical Co., Ltd.), 150 mg flurbiprofen ester (Beijing Tide Pharmaceutical Co., Ltd.), and 15 mg tropisetron (2 mL/hour fixed flow).

### 2.6. Detection of Lymphocyte Subsets

For the three groups of patients, we compared the percentages of peripheral NK cells and T cell subsets, including CD3+% (percentage of total T cells), CD4+% (percentage of helper T cells), CD8+% (percentage of inhibitory/cytotoxic cells), and CD4+/CD8+ ratio at 6:00 am the next day after the first hospital admission, at 6:00 am on the day of surgery, at the time of extubation, and at 6:00 am the next day after surgery. The blood sample was centrifuged at 4°C, 3000 rpm, for 10 min, to separate the red blood cells, buffy coat, and plasma. Then, the plasma was separated, and the buffy coat was entirely taken for flow cytometry. T lymphocyte subsets and NK cells were evaluated in peripheral venous blood using flow cytometry (BD FACSCalibur flow cytometer; BD Biosciences). The forward scatter (FSC) and side scatter (SSC) of the excitation light reflected the cell size and internal structure, respectively. The distributions and quantities of lymphocytes expressing CD3 (mature T cells), CD4 (helper and regulatory T cells), and CD8 (cytotoxic T cells) were evaluated using specific antibodies (BD Lymphocyte Subset Detection Reagent and BD NK Cell Detection Reagents) and immunofluorescence. The CellQuest software was used for acquiring and analyzing data.

### 2.7. Clinical Data

The following data were recorded: age, BMI, operative time, intraoperative blood loss, and infusion volume. The percentages of NK cells, CD3^+^ cells, CD4^+^ cells, and CD8^+^ cells (NK%, CD3%, CD4%, and CD8%, respectively) and the ratio of CD4^+^ cells to CD8^+^ cells (CD4/CD8 ratio) in peripheral venous blood were determined at four time points: the day after the first admission; 06:00 on the day of surgery; immediately after removal of the tracheal tube; and 06:00 on the day after surgery. The main outcome measures were postoperative NK%, CD3%, CD4%, CD8%, and CD4/CD8 ratio.

### 2.8. Sample Size Calculation

The patients in this study had been recruited in a clinical trial (NCT03872635), assessing the effects of preoperative oral carbohydrate on insulin resistance and immune cell levels after surgery. Sample size calculation in the original trial was based on a preliminary assessment of blood glucose levels at 24 hours after surgery; the values were 6.00 ± 3.40 mmol/L in patients treated with surgery only, 5.07 ± 5.25 mmol/L in patients treated with NAC and surgery, and 7.32 ± 2.35 mmol/L (*n* = 10 per group) in patients treated with NAC, preoperative oral carbohydrate, and surgery. The minimum sample size was calculated using the following formula:

(1) $$\begin{array}{c} n=\varphi^{2}\frac{\left(\sum {\text{si}}^{2}/k\right)}{\sum {\left(\text{xi}-\text{xmean}\right)}^{2}/\left(k-1\right)}. \end{array}$$

The parameters are as follows: (1) *α* = 0.05; (2) *β* = 0.10; (3) *k* is number of groups; (4) *φ* is, in this example, where *k* = 3, degrees of freedom of *V*1 = *k* − 1 = 2, degrees of freedom of *V*2 = *N* − 1, *N* was unknown and could take a maximum of ∞, and using a look-up table, *φα*, *β*, *k*-1, ∞ = 2.52; (5) xi and si were, respectively, the mean of the *i* group (x1 = 6.00, x2 = ⋯) and the standard deviation of the *i* group (s1 = 3.40, s2 = ⋯); and (6) determination of xmean is xmean = (*x*1 + *x*2 + *x*3)/*k*.

The minimum sample size was calculated to be 74 and was expanded by 10% to take into account the loss to follow-up. Therefore, 81 patients (27 per group) were enrolled.

### 2.9. Statistical Analysis

The analysis was performed using SPSS 22.0 (IBM Corp., Armonk, NY, USA). Continuous data are expressed as the mean ± standard deviation. Normally distributed continuous data were compared between using the independent samples *t*-test (two groups) and using one-way analysis of variance (ANOVA; three groups). Nonnormally distributed continuous data were compared between groups using the rank-sum test. Intragroup comparisons between different time points were made using repeated measures ANOVA. The chi-squared test was used for the comparisons of count data. *P* < 0.05 was considered statistically significant.

## 3. Results

### 3.1. Baseline Clinical Characteristics of the Study Participants

**Figure 1:**
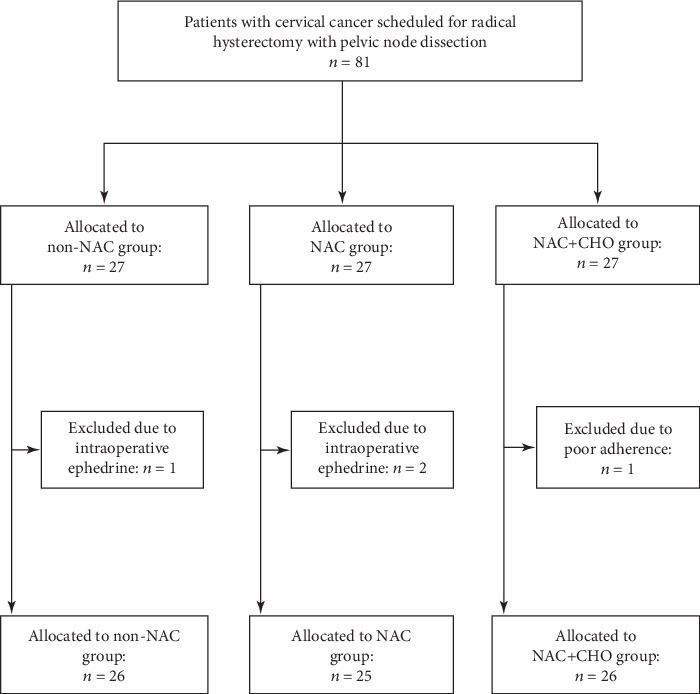
Flow chart showing patient enrollment in the study. CHO: carbohydrate; NAC: neoadjuvant chemotherapy.

Among the 81 patients initially enrolled in the study (27 in each group), one patient in the non-NAC group and two patients in the NAC group were excluded due to the intraoperative administration of ephedrine, and one patient in the NAC+CHO group was excluded due to poor adherence to the study protocol (i.e., preoperative fasting and consumption of the oral carbohydrate drink). Therefore, the final analysis included 77 patients, with 26 in the non-NAC group, 25 in the NAC group, and 26 in the NAC+CHO group (Figure 1).

The baseline characteristics of the patients in each group are presented in Table 1. There were no significant differences between groups in patient age, BMI, operative time, intraoperative blood loss, and infusion volume (Table 1).

### 3.2. NK% in Peripheral Venous Blood

There were no significant differences between groups in NK% in peripheral venous blood on the day after the first admission and preoperatively on the day of surgery (Table 2). When compared with the preoperative value, NK% was significantly lower after tracheal tube removal and on the day after surgery in all three groups (*P* < 0.05; Table 2). Notably, when compared with the other two groups, the NAC+CHO group had significantly higher NK% after tracheal tube removal and on the day after surgery (*P* < 0.05; Table 2). Indeed, NK% on the day after surgery was similar to that on the day after the first admission in the NAC+CHO group but significantly lower than that on the day after the first admission in the other two groups (Table 2).

### 3.3. CD3% in Peripheral Venous Blood

CD3% (the percentage of lymphocytes that were mature T cells) in peripheral venous blood was similar between the three groups on the day after the first admission and preoperatively on the day of surgery (Table 3). All three groups showed significant declines in CD3% after surgery (*P* < 0.05; Table 3). However, CD3% on the day after surgery was significantly higher in the NAC+CHO group than in the NAC group or non-NAC group (*P* < 0.05; Table 3).

### 3.4. CD4% in Peripheral Venous Blood

As shown in Table 4, CD4% (the percentage of cells that were helper and regulatory T cells) in peripheral venous blood was not significantly different between groups at the preoperative time points, but all three groups exhibited a lower CD4% after surgery (*P* < 0.05). Furthermore, CD4% on the day after surgery was significantly higher in the NAC+CHO group than in the NAC group or non-NAC group (*P* < 0.05; Table 4).

### 3.5. CD8% in Peripheral Venous Blood

As detailed in Table 5, there were no significant differences between groups in CD8^+^ (the percentage of cells that were cytotoxic T cells) on the day after the first admission, although CD8% was higher in the non-NAC group than in the other two groups just before surgery due to a fall in CD8% in the NAC and NAC+CHO groups (*P* < 0.05). Postoperatively, there were significant increases in CD8% in the non-NAC and NAC groups (*P* < 0.05), whereas no such increase occurred in the NAC+CHO group (Table 5).

### 3.6. CD4^+^/CD8^+^ Ratio in Peripheral Venous Blood

Table 6 demonstrates that there were no significant differences between the three groups in the CD4^+^/CD8^+^ ratio on the day after the first admission. However, the CD4^+^/CD8^+^ ratio was significantly lower in the non-NAC group than in the other two groups just before surgery (*P* < 0.05), reflecting the decline in CD8% in the NAC and NAC+CHO groups at this time point (see above). Compared with the value just before surgery, the non-NAC and NAC groups exhibited significant decreases in the CD4^+^/CD8^+^ ratio (*P* < 0.05), whereas little or no change was observed in the NAC+CHO group (Table 6).

## 4. Discussion

A notable finding of this study was that women with cervical cancer exhibited decreases in NK%, CD3%, and CD4% and an increase in CD8% after RH with PLND. However, the changes in NK%, CD3%, CD4%, and CD8% were less pronounced in the NAC-CHO group than in the NAC and non-NAC groups. These data suggest that preoperative oral carbohydrate might help to reduce disturbances in immune cell numbers in patients treated surgically for cervical cancer.

Antitumor immunity mainly relies on the cellular immune system, including T cell subsets and NK cells [29]. T_H_1 helper cells exert antitumor effects via recruitment of CD8^+^ cytotoxic T cells, NK cells, and macrophages that kill cancer cells. T_H_2 helper cells have antitumor actions through the recruitment of eosinophils [29]. CD8^+^ cytotoxic T cells and NK cells can target and kill tumor cells [29]. By contrast, CD4^+^ regulatory T cells suppress antitumor immunity [29]. Cancer cells express various cytokines that inhibit lymphocyte differentiation/proliferation, reduce CD4^+^ cell levels, and increase CD8%, which further inhibits CD4^+^ T lymphocytes in a vicious circle [30]. This suppression of normal immune responses facilitates tumor growth and proliferation [12]. With regard to cervical cancer, previous research has revealed changes in T cell subsets and NK cell populations as well as inhibition of NK cell activity by CD4^+^ regulatory T cells [13–15]. Thus, it is important that disturbances in immune function are minimized during the treatment of cervical cancer.

NAC can decrease cervical tumor size, improve surgical feasibility, improve progression-free survival and overall survival, and reduce the requirement for postoperative adjuvant therapy [6–11, 31]. Paclitaxel and cisplatin selectively kill tumor cells and may improve immune status in patients with cancer [32, 33]. Some studies have observed no effects of NAC on lymphocyte numbers in patients with cervical cancer [17, 18]. However, chemotherapy has been reported to inhibit NK cell activity [19]. In the present study, NK%, CD3%, and CD4% were not significantly different between the three groups at both preoperative time points. This suggests that the effects of NAC on NK%, CD3%, and CD4% were either negligible or short-lasting (i.e., <4 weeks). However, preoperative CD8% was lower in the NAC groups than in the non-NAC group. This suggests a possible effect of NAC on CD8^+^ lymphocyte numbers, as reported previously in an animal study [34]. If so, NAC might be a “double-edged sword” in that it not only targets tumor cells but also reduces cytotoxic T cell function and thus antitumor immunity.

Surgery can cause an immunosuppressive effect in the early postoperative stage due to surgical stress and trauma, effects of general anesthetic agents, intraoperative hypothermia, intraoperative blood loss and transfusion, and hyperglycemia due to postoperative insulin resistance [35–37]. Major surgery can impair cell-mediated immunity in the short term, with decreases in tumor necrosis factor-alpha, interleukin-2, interferon-gamma, and lymphoproliferation [38]. Consistent with our findings, a previous investigation reported that RH for cervical cancer reduced CD4^+^ T cell and NK cell numbers [16]. It is possible that immune dysfunction after surgical treatment of cervical cancer may impair antitumor immunity.

Preoperative oral carbohydrate can attenuate postoperative reductions in T cell and NK cell numbers [23]. We also found that a carbohydrate drink given 2 hours before surgery limited the postoperative changes in NK%, CD3%, and CD4%. Preoperative oral carbohydrate may have several benefits in patients undergoing major surgery, including a reduction in postoperative insulin resistance and blood glucose levels [21–28]. In healthy people, hyperglycemia is associated with decreased lymphocyte counts [39]. Furthermore, severe perioperative hyperglycemia can suppress T cell activation, basophil count, and monocyte function [40]. We speculate that the beneficial effects of preoperative oral carbohydrate on postoperative NK%, CD3%, and CD4% were due to suppression of perioperative insulin resistance and hyperglycemia. However, additional studies are needed to establish the validity of this hypothesis.

The benefit of oral carbohydrates on tumor immunity before surgery is mainly related to increased blood glucose levels. The effect of blood glucose levels on the immune system is also reflected in the production of lymphocytes. Indeed, in healthy subjects, in the state of hyperglycemia, the lymphocytes and their subpopulations are decreased, while the abnormalities decrease when the blood glucose decrease [41]. At the same time, hyperglycemia is closely related to the increase of cytotoxic T cells and the decrease of immune T cells. The negative regulation of cell activity during hyperglycemia is due to the decrease in CD4+/CD8+% and the enhanced expression of HLA-DR antigens [42], thus ensuring the stability of serum environmental glucose levels and possibly can avoid the interference with normal cells to execute normal activities, thereby ensuring their self-regulating function, shortening the inflammatory processes, and playing a positive effect on the body's immunity. Nevertheless, the exact mechanisms remain to be determined.

This study has some limitations. This was a single-center study. The different tumor types and chemotherapy regimens introduced some biases in the analyses. Neither the patients nor investigators were blinded to the intervention, which may have introduced bias. In addition, the generalizability of the findings is unknown. The sample size was small, so the study may have been underpowered to detect some real differences between groups. The non-NAC group enrolled patients with a different disease stage to those in the NAC groups, which may have introduced bias. The patients were followed up for only 1 day after surgery, so longer-term effects were not determined. We did not perform a safety analysis.

## 5. Conclusion

Preoperative oral carbohydrate treatment may help to attenuate the changes in NK and T cell populations after treatment of cervical cancer by NAC and surgery. However, large-scale, multicenter, randomized controlled trials are required to validate our findings and establish whether preoperative oral carbohydrate might reduce disease recurrence or improve survival rate.

## Figures and Tables

**Table 1:** Baseline clinical characteristics of the study participants in the three groups.

Characteristic	Non-NAC group (*n* = 26)	NAC group (*n* = 25)	NAC+CHO group (*n* = 26)	*F*	*P*
Age (years)	52.92 ± 7.56	50.08 ± 7.87	53.65 ± 8.40	1.458	0.239
Body mass index (kg/m^2^)	21.03 ± 1.38	20.99 ± 0.99	20.60 ± 1.22	1.011	0.369
Operative duration (min)	180.50 ± 19.19	188.01 ± 24.48	187.74 ± 17.89	1.093	0.341
Blood loss (mL)	211.7 ± 30.09	214.65 ± 34.64	221.54 ± 36.23	0.572	0.567
Infusion volume (mL)	1846.8 ± 267.54	1816.03 ± 339.81	1803.93 ± 346.44	0.124	0.883

Data are presented as the mean ± standard deviation.

**Table 2:** Percentage of natural killer (NK) cells in peripheral venous blood.

Time point	Non-NAC group	NAC group	NAC+CHO group	*F* ^2^	*P* ^2^
Day after first admission	14.29 ± 8.39	13.38 ± 6.42	12.84 ± 9.10	0.213	0.809
Before surgery	15.11 ± 3.23	16.17 ± 4.73^1^	17.42 ± 3.65^a,1^	2.261	0.111
After tracheal tube removal	5.93 ± 2.84^1,2^	7.05 ± 4.52^a,1,2^	12.73 ± 5.12^a,b,2^	104.388	0.000
Day after surgery	4.91 ± 1.48^1,2^	5.83 ± 2.93^a,1,2,3^	12.24 ± 5.71^a,b,2^	233.649	0.000
*F* ^1^	35.821	44.839	5.942		
*P* ^1^	0.000	0.000	0.011		

^1^ *P* < 0.05 vs. day after the first admission; ^2^*P* < 0.05 vs. before surgery; ^3^*P* < 0.05 vs. after tracheal tube removal; ^a^*P* < 0.05 vs. the non-NAC group; ^b^*P* < 0.05 vs. the NAC group.

**Table 3:** Percentage of CD3^+^ cells (all T cells) in peripheral venous blood.

Time point	Non-NAC group	NAC group	NAC+CHO group	*F* ^2^	*P* ^2^
Day after first admission	71.21 ± 9.88	72.63 ± 7.38	73.16 ± 6.83	0.398	0.673
Before surgery	71.56 ± 11.70	76.21 ± 12.62	79.35 ± 17.46	1.961	0.148
After tracheal tube removal	63.84 ± 6.72^1,2^	66.78 ± 13.34^1,2^	73.29 ± 25.94^1,2^	2.036	0.138
Day after surgery	64.58 ± 5.26^1,2^	69.42 ± 14.08^1,2^	76.39 ± 23.82^a,1,2^	3.464	0.036
*F* ^1^	6.080	2.920	0.522		
*P* ^1^	0.001	0.048	0.608		

^1^ *P* < 0.05 vs. day after the first admission; ^2^*P* < 0.05 vs. before surgery; ^3^*P* < 0.05 vs. after tracheal tube removal; ^a^*P* < 0.05 vs. the non-NAC group; ^b^*P* < 0.05 vs. the NAC group.

**Table 4:** Percentage of CD4^+^ cells (helper and regulatory T cells) in peripheral venous blood.

Time point	Non-NAC group	NAC group	NAC+CHO group	*F* ^2^	*P* ^2^
Day after first admission	41.53 ± 6.96	44.87 ± 9.16	43.0 ± 10.25	0.918	0.404
Before surgery	42.00 ± 7.66	49.49 ± 5.57^a,1^	49.43 ± 2.84^a,1^	14.807	0.502
After tracheal tube removal	35.71 ± 3.98^1,2^	32.86 ± 6.95^1,2^	39.87 ± 7.74^a,b,2^	7.913	0.001
Day after surgery	32.00 ± 3.97^1,2,3^	29.73 ± 3.03^1,2^	37.99 ± 5.15^a,b,1,2^	27.544	0.000
*F* ^1^	14.776	51.708	50.979		
*P* ^1^	0.000	0.000	0.000		

^1^ *P* < 0.05 vs. day after the first admission; ^2^*P* < 0.05 vs. before surgery; ^3^*P* < 0.05 vs. after tracheal tube removal; ^a^*P* < 0.05 vs. the non-NAC group; ^b^*P* < 0.05 vs. the NAC group.

**Table 5:** Percentage of CD8^+^ cells (cytotoxic T cells) in peripheral venous blood.

Time point	Non-NAC group	NAC group	NAC+CHO group	*F* ^2^	*P* ^2^
Day after first admission	25.56 ± 7.49	24.01 ± 6.58	25.88 ± 9.36	0.418	0.660
Before surgery	24.75 ± 3.42	19.54 ± 5.38^a,1^	18.51 ± 5.59^a,1^	11.508	0.000
After tracheal tube removal	30.63 ± 3.51^1,2^	25.97 ± 7.49^a,2^	19.40 ± 2.58^a,b,1^	33.018	0.000
Day after surgery	32.92 ± 3.91^1,2^	28.88 ± 2.94^a,2^	22.20 ± 6.69^a^	40.844	0.000
*F* ^1^	15.853	6.077	5.040		
*P* ^1^	0.000	0.000	0.008		

^1^ *P* < 0.05 vs. day after the first admission; ^2^*P* < 0.05 vs. before surgery; ^3^*P* < 0.05 vs. after tracheal tube removal; ^a^*P* < 0.05 vs. the non-NAC group; ^b^*P* < 0.05 vs. the NAC group.

**Table 6:** CD4^+^/CD8^+^ ratio in peripheral venous blood.

Time point	Non-NAC group	NAC group	NAC+CHO group	*F* ^2^	*P* ^2^
Day after first admission	1.75 ± 0.54	2.09 ± 1.05	0.97 ± 0.99	0.998	0.375
Before surgery	1.73 ± 0.44	2.75 ± 0.80^a,1^	2.78 ± 1.39^a,1^	19.585	0.000
After tracheal tube removal	1.11 ± 0.16^1,2^	2.10 ± 0.70^a,2^	2.21 ± 1.04^a^	0.766	0.459
Day after surgery	0.91 ± 0.17^1,2^	2.09 ± 0.40^a,2^	2.42 ± 0.73^a,b^	20.768	<0.001
*F* ^1^	38.657	4.501	2.436		
*P* ^1^	0.000	0.013	0.090		

^1^ *P* < 0.05 vs. day after the first admission; ^2^*P* < 0.05 vs. before surgery; ^3^*P* < 0.05 vs. after tracheal tube removal; ^a^*P* < 0.05 vs. the non-NAC group; ^b^*P* < 0.05 vs. the NAC group.

## Data Availability

The datasets used and/or analyzed during the current study are available from the corresponding author on reasonable request.
